# Body Height Estimation According to Deciduous Dental Crown Height in a Peruvian Sample of Preschool Children

**DOI:** 10.1155/2024/3664231

**Published:** 2024-05-14

**Authors:** Aldana Yanira Pimentel-Chalco, Kilder Maynor Carranza-Samanez, Julissa Amparo Dulanto-Vargas

**Affiliations:** ^1^School of Dentistry, Universidad Científica del Sur, Lima, Peru; ^2^Research Group in Dental Sciences, School of Dentistry, Universidad Científica del Sur, Lima, Peru

## Abstract

**Introduction:**

Odontometry and body height are distinctive biological traits, making their relationship relevant in the identification of individuals. The objective of this study was to estimate body height according to the height of the crown of deciduous teeth in Peruvian preschool children.

**Materials and Methods:**

This analytical study was applied to a calculated sample of 204 preschoolers between 3 and 5 years of age (34 per sex/age group) with fully erupted upper anterior deciduous teeth (from #53 to #63). Measurements included body height with a stadiometer (106 ± 6.56 cm; 94–123 cm) and crown height from canine to canine (3.06 mm in #62 to 8.13 mm in #53) in models with digital vernier (intraclass correlation coefficient ≥ 0.781). Linear regression models included calculation of the regression coefficient (*β*) to predict height based on crown height for each deciduous tooth at a significance level of *P* < 0.05.

**Results:**

There was no correlation between body height and crown height by tooth type in the total sample (*P* ≥ 0.05), but there was in a 3-year-old female in #52 (*r* = 0.4: moderate) and a 5-year-old female in #53 and #63 (*r* = 0.36–0.38: low) (*P* < 0.05). Body height prediction equations are shown according to crown height per tooth and sex/age groups. The regressions were significant, explaining 13%–18% of 3-year-old females in #53 and #52 (1.85–1.86 cm error) and 5-year-old females in #53, #51, and #63 (4.61 at 4.63 cm error) (*P* < 0.05).

**Conclusions:**

The odontology method using crown height of the upper anterior deciduous teeth estimated body height in Peruvian girls of 3 and 5 years of age. The teeth are resistant to traumatic forces so these could be used as a body height estimation parameter for forensic human identification.

## 1. Introduction

Teeth are important anatomical structures in forensic, genetic, anthropological, and odontogenic sciences [1, 2]. They are frequently used to identify decomposed corpses in mass disasters [3] because they are resistant to tissue loss due to degradation and high temperatures [4–6]. They are the most stable tissues in the human body unlike soft tissues that are highly susceptible to decomposition [1, 4, 7].

Human dentition is not the same between two individuals due to morphology and innate placement. A dental impression replicates the dental arch and being useful for dental registration [4, 8]. Individual characteristics and the resistance to postmortem teeth alterations are currently a cornerstone for dental identification in legal dentistry [9, 10].

Dental morphometry studies the normal anatomy and structure, with odontometry being more precise, evaluating anatomical dimensions through measurements of length, mesiodistal, and labiolingual diameter of the clinical crown [11–14]. This quantitative technique is of interest for the analysis of tooth shape and size compared to other variables of interest, such as body height [1–3, 7, 15].

Age, sex, and ethnicity are key features in forensic identification [3, 16], in addition to body height, that is, the height of an individual in an upright vertical position [2, 17, 18] because skeletal remains enable the distinction between individuals [3, 19]. This variable is directly related to craniofacial bone growth [20] which some authors correlate with tooth length [12, 15].

Body height is estimated from bony parts [3, 7, 12, 18, 21–23]; however, there is limited literature on the prediction of height [1, 2] from teeth [7, 11, 15]. Some studies used a predictive formula for height from the mesiodistal diameter of incisors and canines [22, 23], while others related body height with the crown height of deciduous teeth [2, 7, 20]. However, this relationship may be influenced by ethnicity [21].

The determination of height is important in the medicolegal identification process [12] and is especially useful in a context in which skeletal remains undergo a taphonomic process and should also be estimated considering ethnicity and age. Therefore, the aim of this study was to estimate height according to deciduous dental crown height in a Peruvian sample of preschool children.

## 2. Materials and Methods

### 2.1. Study Design and Ethics

This cross-sectional analytical study was approved by the Institutional Research Ethics Committee of the Universidad Científica del Sur in Lima—Peru (N°241-CIEI-CIENTÍFICA-2022) and performed following the STROBE guidelines (Table S1) and the principles of the Declaration of Helsinki. Authorization was obtained from the educational institutions and informed consent was obtained provided by the parents/guardians and the children at inclusion.

### 2.2. Sample Selection

From a population of 590 preschoolers from five primary schools (two public (81.4%) and three private (18.6%)) in the city of Lima (Peru) enrolled in the year 2022 (Source: ESCALE—Government of Peru), a sample of 204 children of 3–5 years of age with all the upper anterior deciduous teeth fully erupted (#51, #52, #53, #61, #62, and #63) was recruited. The sample was selected at convenience until completing six groups according to age and sex (34 in each group). The exclusion criteria were (i) children with a fractured, cavitated upper anterior tooth, abnormal morphology, or excessive wear, (ii) children who could not tolerate dental impression, and (iii) models with defects or bubbles preventing coronal measurement. The sample size was calculated with Epidat v.4.2. software according to the correlation coefficient formula based on data from a previous study [1] in which the lowest correlation was 0.174 (#63 in female), with a 95% confidence level (CI) and 80% power.

### 2.3. Pilot Study and Calibration

A pilot study in 20 preschool children (10% of the sample) was carried out in order to standardize body height and the tooth measurement procedure. The principal investigator (AYPC) was trained and calibrated by a forensic expert (KMCS). The intraclass correlation coefficient (ICC) of crown height of total teeth was “good” according to the Landis and Koch criteria for inter-examiner agreement (ICC = 0.781 (95% CI: 0.639−0.862)) and for intraexaminer agreement with a 2-week interval between reevaluation (ICC = 0.873 (95% CI: 0.818−0.912)). The pilot sample was included in the study.

### 2.4. Body Height and Crown Height

**Figure 1:**
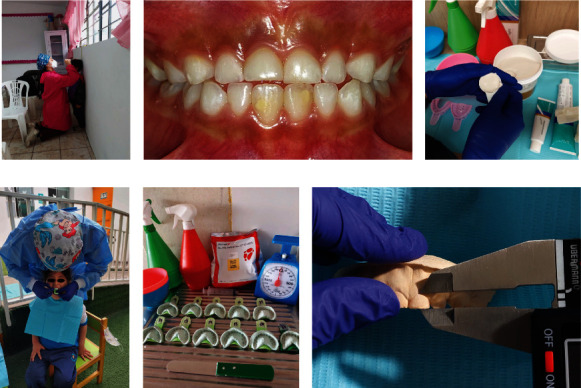
Study procedures: (a) measurement of height, (b) anterosuperior dental sector, (c) impression materials and preparation of the mold, (d) impression taking, (e) casting of models, and (f) measurement of crown heights.

Sex and age data were verified in the school registry. A universal measuring tape was used to measure the body height of preschool children in centimeter after removing footwear and standing upright against the flat surface of a classroom wall [1, 12, 20]. Then, a maxillary canine-to-canine impression was taken with a plastic partial tray using silicone impression material mixed for 30 s (Speedex® Trial Kit, Coltene, Switzerland) with rapid setting time (1 min 30 s). Next, the dental impression was disinfected with sodium hypochlorite (1%: 10 s), washed with water (10 s), dried naturally with absorbent paper, and stored in individual airtight containers. After 2 hr, casting was carried out with type IV dental plaster (Elite Rock® Sandy Brown, Zhermack, Italy) according to the standard procedure (water/powder ratio: 20 ml/100 g). Demolding was performed 1 hr later and the height of the dental crown was measured using a calibrated vernier instrument (Ubermann®, Chile). The height was recorded in millimetre as the distance from the incisal edge or vestibular cusp to the highest gingival line of the vestibular surface of the crown. Impressions and measurements were made by a single observer (AYPC) (Figure 1).

### 2.5. Data Analysis

The descriptive analysis included mean, standard deviation, minimum, maximum, coefficient of determination (*R*^2^), and standard error of the estimation. A normal distribution was corroborated with the Shapiro–Wilk test. Inferential statistics were performed with the ANOVA test with post hoc Bonferroni (equal variances) and Tamhane (nonequal variances). The linear regression models included the calculation of the regression coefficient (*β*) for the prediction of body height based on crown height per deciduous tooth. Data were analyzed with IBM-SPSS v26 statistical software (SPSS Inc., Chicago, IL, USA) with a 95% CI.

## 3. Results

The body height of the preschool children aged 3–5 years ranged from 94 to 123 cm with a mean of 106 ± 6.56 cm and with values from higher to lower in groups of 5 years of age in both sexes, >4 years of age in both sexes, >3-year-old males, and >3-year-old females (*P*=0.001) (Table 1).

The average crown heights of the six deciduous teeth in groups by sex and age are presented in Table 2. Tooth crown heights in the sample ranged from 3.06 (#62) to 8.13 mm (#53). There was symmetry of heights by homologous teeth in all sex and age groups (*P* ≥ 0.05) being greater in canines (5.48 ± 0.68 mm), central incisors (5.44 ± 0.67 mm), and lateral incisors (4.93 ± 0.60 mm) in the total sample (*P*=0.001).

**Figure 2:**
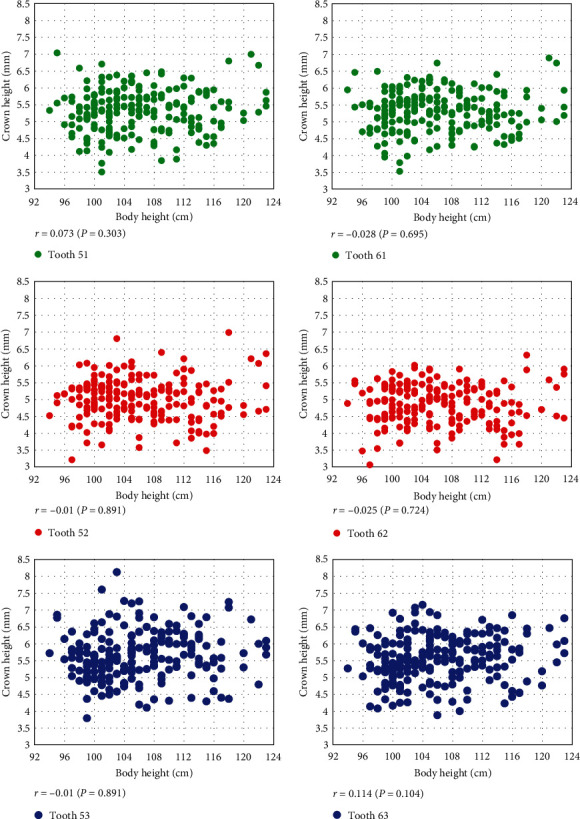
Correlation of body height and crown height according to deciduous tooth in the total sample.

The scatter plot shows that body and coronal height were not correlated by tooth type (*P* ≥ 0.05) (Figure 2), but there was a significant positive correlation (*P* < 0.05) in 3-year-old females in #52 (*r* = 0.4: moderate) and 5-year-old females in #53 and #63 (*r* = 0.36–0.38: low) (Table 3).

The body height prediction equations according to crown height for each tooth and sex and age groups are shown in Table 4.

The body height regressions were statistically significant, explaining the differences of 13%–18% in 3-year-old females in #53 and #52 (1.85–1.86 cm error) and 5-year-old females in #53, #51, and #63 (4.61–4.63 cm error) (*P*  < 0.05) (Table 5).

## 4. Discussion

Body and tooth morphometry are relevant for human identification in forensic science. In complex cases, the greater the number of parameters pointing to accurate identification, the more socially useful they will be from a legal point of view [24]. While studies have been carried out to predict body height through the length of the deciduous dental crowns in adults, none have been performed in Peruvian children. The present study carried out this estimation by finding a correlation between body height and crown height in 3-year-old girls in left lateral canines and incisors and 5-year-old girls in left canines and central incisors. Linear regression analysis established predictive equations for body height based on crown height that were found to be significant.

The body height of the preschool children studied ranged from 94 to 123 cm with a mean of 106 cm, being similar according to sex at 4 and 5 years of age and greater in males than females at 3 years of age. From the analysis of the body height-for-age tables of the World Health Organization, we inferred that no child had a low body height for age (<percentile 3), and rather most were within the norm and some even presented a higher body height (>percentile 97). Growth is related to environmental and genetic factors and was therefore relevant to characterize the preschoolers evaluated in this study. Most of the children were from public schools in districts of Lima (0 m above sea level) with high population density located within the 25% of the less poor districts of Peru (INEI: Peru poverty map, 2018). The results are of note in relation to Peruvian public health surveillance, showing that the height of the children in the present study was 0.39–3.96 cm greater than that of 4- and 5-year-old Peruvian schoolchildren reported in 2011 [25].

The deciduous anterior coronal height was described in this study, demonstrating its bilateral symmetry, similarity by sex and accentuated differences at older ages between canines (5.24–6.07 cm) and central incisors (5.11–5.53 cm) versus lateral incisors (4.77–5.14 cm). A recent review reported that this variable was analyzed in an Asian population [13] of 250 children aged 3–6 years and in four studies in Saudi Arabia [1] and India [2, 7, 15], in which the heights were presented only by sex (not by age) and measured only teeth of one hemi-arch [2, 7, 15]. In two of the studies from India [2, 15], the crown heights showed a trend to be higher than what was found in the present study. However, the similar proportion by sex [1, 2, 7] and differential by tooth type [1, 2, 7, 15] were compatible with our study. Possible explanations for our results suggest that the growth of the children studied was not influenced by relevant environmental factors that may generate asymmetries, and that differential chromosomal expression is unusual in teeth of early development, presenting a reduction in the height of the lateral incisors which are teeth of variable development [26].

The correlation of crown and body height in this study was not corroborated in the total sample but was corroborated when differentiated by sex. Significant predictions were tested in 3-year-old (tooth #52 and #53) and 5-year-old girls (tooth #53, #51, and #63), although their explanatory power did not exceed 18% of the sample. Certain body heights were also similarly predicted based on #52 crown heights in girls [2, 15]. In contrast to this study, other authors did not find association [1] or also found them in males in #51 and #53 [7]. These previous studies evaluated a different population (Asian) and had a limited sample size (30–100 individuals) and used predictive formulas that did not consider age. The differences between the present study and the Asian studies are that ours included a larger sample of South American children (*n* = 204) and included results by sex and age groups. We consider the inclusion of time as a biological variable to reduce the error in the equations for the identification of individuals. It should also be considered that the coronal trait involves embryonic odontogenesis, especially of the dentin layer, and thus, coronal height is more associated with genetics, unlike body height, which is greatly influenced by nutrition [25, 26].

Statistical predictions based on deciduous crown heights could help other methods in the accuracy of identification of preschool children in a specific sample of Peru. The present study established a proportional sample by sex and age, which allowed reducing the margin of error. These results were established in a specific Peruvian sample, and thus, in order to extrapolate the formulas to the general population, it is necessary to involve participants from other regions.

## 5. Conclusions

Within the limitations of the present study, it was concluded that the odontology method that considered the crown height of the upper anterior deciduous teeth as a reference estimated the body height in Peruvian girls of 3 and 5 years. The teeth are resistant to degradation and high temperatures so these could be used as a body height estimation parameter for forensic human identification as in cases of mass disasters.

## Figures and Tables

**Table 1:** Differentiation of body height according to sex and age groups in a sample of Peruvian preschool children.

Sex and age groups	Body height (cm)					
	*n*	Mean	SD	Minimum	Maximum	*P* value
Males 3 years of age	34	102.00^A^	2.81	95	107	0.001 ^*∗∗∗*^
Males 4 years of age	34	105.09^B^	4.52	95	115	
Males 5 years of age	34	111.65^C^	5.61	98	123	
Females 3 years of age	34	99.24^D^	1.95	94	102	
Females 4 years of age	34	104.76^AB^	4.88	98	115	
Females 5 years of age	34	113.26^C^	4.89	105	123	
Total	204	106.00	6.56	94	123	

Different capital letters indicate significant differences by columns. ANOVA test with Tamhane post-hoc.  ^*∗∗∗*^*P*  < 0.001.

**Table 2:** Comparison of crown height per deciduous tooth according to sex and age groups in a sample of Peruvian preschool children.

Age and sex	Crown height (cm) (mean ± SD)							
	*n*	#53	#52	#51	#61	#62	#63	*P* value
3-year-old males	34	5.38 ± 0.6^Aa^	4.98 ± 0.49^AB^	5.27 ± 0.65^ABa^	5.28 ± 0.54^AB^	4.88 ± 0.5^B^	5.35 ± 0.61^Aac^	0.001 ^*∗∗*^
4-year-old males	34	6.07 ± 0.77^Ab^	5.14 ± 0.68^BC^	5.53 ± 0.54^BDa^	5.44 ± 0.65^BD^	4.94 ± 0.67^C^	5.85 ± 0.72^ADb^	0.001 ^*∗∗∗*^
5-year-old males	34	5.82 ± 0.7^Aab^	5.1 ± 0.7^BC^	5.48 ± 0.69^ABa^	5.38 ± 0.57^ABC^	4.92 ± 0.68^C^	5.64 ± 0.59^Aab^	0.001 ^*∗∗∗*^
3-year-old females	34	5.34 ± 0.51^Aa^	4.96 ± 0.6^AB^	5.11 ± 0.55^ABa^	5.19 ± 0.56^AB^	4.84 ± 0.61^B^	5.24 ± 0.55^ABa^	0.004 ^*∗∗*^
4-year-old females	34	5.73 ± 0.69^ADab^	4.96 ± 0.56^BC^	5.37 ± 0.67^ABDa^	5.36 ± 0.63^AB^	4.81 ± 0.5^C^	5.8 ± 0.58^Dbc^	0.001 ^*∗∗∗*^
5-year-old females	34	5.54 ± 0.76^Aa^	4.9 ± 0.6^B^	5.19 ± 0.69^ABa^	5.18 ± 0.61^AB^	4.77 ± 0.59^B^	5.41 ± 0.77^Aab^	0.001 ^*∗∗∗*^
*P* value		0.001 ^*∗∗∗*^	0.577	0.046 ^*∗*^	0.363	0.821	0.001 ^*∗∗∗*^	

Different capital letters indicate significant differences by row. Different lower case letters indicate significant differences by columns. ANOVA test with Bonferroni post hoc.  ^*∗*^*P* < 0.05,  ^*∗∗*^*P* < 0.01, and  ^*∗∗∗*^*P* < 0.001.

**Table 3:** Correlation of body height and crown height per deciduous tooth according to sex and age groups in a sample of Peruvian preschool children.

Sex and age groups	Correlation coefficient (*r* (*P* value))						
	*n*	#53	#52	#51	#61	#62	#63
Males 3 years of age	34	−0.023 (0.898)	−0.051 (0.773)	0.196 (0.266)	0.158 (0.372)	0.109 (0.539)	0.248 (0.156)
Males 4 years of age	34	0.115 (0.518)	−0.082 (0.646)	−0.119 (0.502)	0.019 (0.914)	−0.094 (0.598)	0.03 (0.865)
Males 5 years of age	34	−0.199 (0.259)	−0.231 (0.188)	−0.165 (0.35)	−0.222 (0.208)	−0.283 (0.105)	−0.128 (0.47)
Females 3 years of age	34	−0.231 (0.189)	0.404 (0.018) ^*∗*^	0.09 (0.614)	0.114 (0.52)	0.121 (0.496)	−0.006 (0.972)
Females 4 years of age	34	0.12 (0.499)	−0.209 (0.236)	0.025 (0.887)	0.021 (0.905)	−0.039 (0.828)	0.004 (0.981)
Females 5 years of age	34	0.38 (0.027) ^*∗*^	0.225 (0.201)	0.265 (0.13)	0.153 (0.386)	0.202 (0.252)	0.359 (0.037) ^*∗*^

*r*, Pearson's correlation.  ^*∗*^*P* < 0.05.

**Table 4:** Mathematical equation for the prediction of body height per deciduous tooth according to sex and age groups in a sample of Peruvian preschool children.

Sex and age groups	Mathematical equation of the body height (cm) = A + *β* (crown height)						
	*n*	#53	#52	#51	#61	#62	#63
Males 3 years of age	34	103.50 – 0.28(*X*)	102.72 – 0.14(*X*)	102.08 – 0.01(*X*)	100.23 + 0.34(*X*)	97.79 + 0.86(*X*)	98.49 + 0.66(*X*)
Males 4 years of age	34	106.26 – 0.19(*X*)	110.56 – 1.07(*X*)	111.26 – 1.12(*X*)	102.96 + 0.39(*X*)	106.47 – 0.28(*X*)	106.05 – 0.16(*X*)
Males 5 years of age	34	121.49 – 1.69(*X*)	116.83 – 1.02(*X*)	117.50 – 1.07(*X*)	125.09 – 2.50(*X*)	121.34 – 1.97(*X*)	118.25 – 1.17(*X*)
Females 3 years of age	34	106.56 – 1.37(*X*)	93.49 + 1.16(*X*)	100.27 – 0.20(*X*)	100.88 – 0.32(*X*)	98.38 + 0.18(*X*)	102.08 – 0.54(*X*)
Females 4 years of age	34	104.39 + 0.07(*X*)	112.37 – 1.53(*X*)	102.41 + 0.44(*X*)	101.91 + 0.53(*X*)	107.80 – 0.63(*X*)	104.58 + 0.03(*X*)
Females 5 years of age	34	100.12 + 2.38(*X*)	102.86 + 2.12(*X*)	100.09 + 2.54(*X*)	101.53 + 2.26(*X*)	104.48 + 1.84(*X*)	98.73 + 2.68(*X*)
Total	204	100.70 + 0.94(*X*)	106.52 - 0.10(*X*)	102.07 + 0.74(*X*)	104.39 + 0.30(*X*)	107.34 - 0.28(*X*)	99.85 + 1.11(*X*)

A, constant; *β*, regression coefficient; and *X*, predictor variable.

**Table 5:** Parameters of the linear regression model for the prediction of body height per deciduous tooth according to sex and age groups in a sample of Peruvian preschool children.

Sex and age groups	#53			#52			#51			#61			#62			#63		
	*R* ^2^	SEE	*P* value	*R* ^2^	SEE	*P* value	*R* ^2^	SEE	*P* value	*R* ^2^	SEE	*P* value	*R* ^2^	SEE	*P* value	*R* ^2^	SEE	*P* value
Males 3 years of age	0.00	2.85	0.729	0.00	2.85	0.887	0.00	2.85	0.985	0.00	2.84	0.717	0.02	2.82	0.383	0.02	2.82	0.419
Males 4 years of age	0.00	4.58	0.854	0.03	4.53	0.363	0.02	4.54	0.455	0.00	4.58	0.752	0.00	4.58	0.815	0.00	4.58	0.884
Males 5 years of age	0.04	5.57	0.233	0.02	5.65	0.472	0.02	5.65	0.460	0.07	5.51	0.145	0.06	5.53	0.177	0.01	5.65	0.491
Females 3 years of age	0.13	1.86	0.039 ^*∗*^	0.13	1.85	0.038 ^*∗*^	0.00	1.98	0.752	0.01	1.98	0.607	0.00	1.98	0.756	0.02	1.96	0.392
Females 4 years of age	0.00	4.96	0.958	0.03	4.88	0.321	0.00	4.95	0.737	0.00	4.94	0.697	0.00	4.95	0.718	0.00	4.96	0.983
Females 5 years of age	0.14	4.61	0.031 ^*∗*^	0.07	4.79	0.139	0.13	4.63	0.037 ^*∗*^	0.08	4.76	0.103	0.05	4.84	0.208	0.18	4.49	0.012 ^*∗*^
Total	0.01	6.54	0.143	0.00	6.57	0.891	0.01	6.56	0.303	0.00	6.57	0.695	0.00	6.57	0.724	0.01	6.53	0.104

*R* ^2^, determination coefficient; SEE, standard error of the estimation.  ^*∗*^*P* < 0.05.

## Data Availability

Data are available upon request.

## Supplementary Materials

Supplementary Materials Table S1: the STROBE statement—checklist of items that should be included in reports of cross-sectional studies.
